# Perceptions of involvement in advance care planning and emotional functioning in patients with advanced cancer

**DOI:** 10.1007/s11764-021-01020-y

**Published:** 2021-04-10

**Authors:** Lente L. Kroon, Janneke van Roij, Ida J. Korfage, An K. L. Reyners, Marieke H. J. van den Beuken-van Everdingen, Marien O. den Boer, Geert-Jan Creemers, Alexander de Graeff, Mathijs P. Hendiks, Jarmo C. B. Hunting, Wouter K. de Jong, Evelien J. M. Kuip, Hanneke W. M. van Laarhoven, Lobke van Leeuwen, Anne S. R. van Lindert, Caroline M. P.W. Mandigers, Peter Nieboer, Annemieke van der Padt-Pruijsten, Tineke J. Smilde, Dirkje W. Sommeijer, Martine F. Thijs, Marian A. Tiemessen, Allert H. Vos, Art Vreugdenhil, Philo T. Werner, Lia van Zuylen, Lonneke V. van de Poll-Franse, Natasja J. H. Raijmakers, on behalf of the eQuiPe study group

**Affiliations:** 1 Department of Research & Development, Netherlands Comprehensive Cancer Organization (IKNL), PO box 19079, 3501 DB Utrecht, The Netherlands; 2 University Medical Center Groningen, University of Groningen, Groningen, The Netherlands; 3 Vrije Universiteit Amsterdam, Amsterdam, The Netherlands; 4 Department of Medical and Clinical Psychology, CoRPS – Center of Research on Psychology in Somatic Diseases, Tilburg University, Tilburg, The Netherlands; 5 Netherlands Association for Palliative Care (PZNL), Utrecht, The Netherlands; 6 Department of Psychology, Pantein, Boxmeer, The Netherlands; 7 Department of Public Health, Erasmus University Medical Center, Rotterdam, The Netherlands; 8 Department of Medical Oncology, University Medical Center Groningen, Groningen, The Netherlands; 9 Center of Expertise Palliative Care, Maastricht University Medical Center, Maastricht, The Netherlands; 10 Department of Medical Oncology, Laurentius Hospital, Roermond, The Netherlands; 11 Department of Medical Oncology, Catharina Hospital, Eindhoven, The Netherlands; 12 Department of Medical Oncology, University Medical Center Utrecht, Utrecht, The Netherlands; 13 Department of Medical Oncology, Northwest Clinics, Alkmaar, The Netherlands; 14 Department of Medical Oncology, St. Antonius Hospital, Utrecht, The Netherlands; 15 Department of Pulmonology, Hospital Gelderse Vallei, Ede, The Netherlands; 16 Department of Medical Oncology, Radboud University Medical Center, Nijmegen, The Netherlands; 17 Department of Medical Oncology, Cancer Center Amsterdam, Amsterdam University Medical Centers, University of Amsterdam, Amsterdam, The Netherlands; 18 Department of Medical Oncology, Diakonessenhuis, Utrecht, The Netherlands; 19 Department of Pulmonology, University Medical Center Utrecht, Utrecht, The Netherlands; 20 Department of Medical Oncology, Canisius Wilhelmina Hospital, Nijmegen, The Netherlands; 21 Department of Medical Oncology, Wilhelmina Hospital Assen, Assen, The Netherlands; 22 Department of Internal Medicine, Maasstad Hospital, Rotterdam, The Netherlands; 23 Department of Medical Oncology, Jeroen Bosch Hospital, ‘s-Hertogenbosch, The Netherlands; 24 Department of Internal Medicine, FlevoHospital, Almere, The Netherlands; 25 Department of Medical Oncology, Ikazia Hospital, Rotterdam, The Netherlands; 26 Department of Pulmonology, Dijklander Hospital, Hoorn, The Netherlands; 27 Department of Medical Oncology, Bernhoven Hospital, Uden, The Netherlands; 28 Department of Medical Oncology, Maxima Medical Center, Eindhoven, The Netherlands; 29 Department of Medical Oncology, VieCuri Medical Center, Venlo, The Netherlands; 30 Department of Medical Oncology, Erasmus Medical Center Cancer Institute, Rotterdam, The Netherlands; 31 Department of Medical Oncology, Amsterdam University Medical Centers, Amsterdam, The Netherlands; 32 Division of Psychosocial Research and Epidemiology, The Netherlands Cancer Institute, Amsterdam, The Netherlands

**Keywords:** Advance care planning, Quality of life, Advanced cancer, Palliative care

## Abstract

**Purpose:**

Advance Care Planning (ACP) is positively associated with the quality of care, but its impact on emotional functioning is ambiguous. This study investigated the association between perceptions of ACP involvement and emotional functioning in patients with advanced cancer.

**Methods:**

This study analyzed baseline data of 1,001 patients of the eQuiPe study, a prospective, longitudinal, multicenter, observational study on quality of care and quality of life in patients with advanced cancer in the Netherlands. Patients with metastatic solid cancer were asked to participate between November 2017 and January 2020. Patients’ perceptions of ACP involvement were measured by three self-administered statements. Emotional functioning was measured by the EORTC-QLQ-C30. A linear multivariable regression analysis was performed while taking gender, age, migrant background, education, marital status, and symptom burden into account.

**Results:**

The majority of patients (87%) reported that they were as much involved as they wanted to be in decisions about their future medical treatment and care. Most patients felt that their relatives (81%) and physicians (75%) were familiar with their preferences for future medical treatment and care. A positive association was found between patients’ perceptions of ACP involvement and their emotional functioning (b=0.162, *p<0.001*, 95%CI[0.095;0.229]) while controlling for relevant confounders.

**Conclusions:**

Perceptions of involvement in ACP are positively associated with emotional functioning in patients with advanced cancer. Future studies are needed to further investigate the effect of ACP on emotional functioning.

**Trial registration number:**

NTR6584 Date of registration: 30 June 2017

**Implications for Cancer Survivors:**

Patients’ emotional functioning might improve from routine discussions regarding goals of future care. Therefore, integration of ACP into palliative might be promising.

## Introduction

Cancer is a major cause of morbidity and mortality worldwide. Despite great improvements in cancer prevention, diagnosis, and treatment, many patients are still being diagnosed with advanced cancer. Patients with advanced cancer experience a decline in emotional functioning as the disease progresses [1]. Improving this aspect of their quality of life (QoL) is essential. Timely integration of palliative care into oncology care increases patients’ QoL and might decrease the risk of depression [1].

An important aspect of palliative care is advance care planning (ACP). ACP is a process in which patients define their goals and preferences for future medical treatment and care, discuss these with family and healthcare providers, and record and review these preferences regularly. ACP discussions are positively associated with receiving appropriate end-of-life care, increased satisfaction with end-of-life care, decreased use of hospital care, and increased use of hospice care [2].

Although palliative care interventions aim to improve patients’ QoL, it remains unclear whether ACP also positively impacts emotional functioning. Brinkman et al. [2] reported inconclusive results regarding the effects of ACP on emotional functioning in their systematic literature review. Recently, a cluster-randomized controlled trial among patients with advanced cancer showed no differences in emotional functioning between the ACP and the control group [3]. So far, no study has investigated the association between patients’ perceptions of involvement in ACP and their emotional functioning. This study aims to assess the association between *perceptions* of ACP involvement and emotional functioning in patients with advanced cancer, without focusing on *actual participation* in ACP conversations.

## Methods

### Study design

A cross-sectional analysis using baseline data of a prospective, longitudinal, multicenter, observational study on quality of care and QoL of patients with advanced cancer and their relatives (eQuiPe) was conducted [4]. The Medical Research Ethics Committee of the Antoni van Leeuwenhoek hospital (METC17.1491) reviewed the study protocol, and it was exempted from full medical ethical review according to the Dutch Medical Research Involving Human Subjects Act WMO.

### Setting and study population

Patients with advanced cancer were recruited in 40 hospitals in the Netherlands between November 2017 and January 2020. Patients were asked to complete a questionnaire regarding quality of care and QoL every 3 months till their death. All adult patients with a diagnosis of metastatic solid cancer (stage IV) who were able to complete Dutch questionnaires were eligible. To limit inclusion of patients with a relatively long prognosis, additional inclusion criteria for breast and prostate cancer were respectively metastases in multiple organ systems and castration-resistant disease.

### Data collection

Patients were screened for eligibility and asked to participate by their physician or were self-enrolled. All eligible patients were contacted by the researcher to discuss participation. In total 1695 eligible patients were contacted by the research team by phone, of which 15% of the patients did not want to participate due to no interest, bad health, too confronting, or lack of time. After giving informed consent, patients received questionnaires on paper or online via the Patient Reported Outcomes Following Initial treatment and Long-term Evaluation of Survivorship (PROFILES) registry [5]. Before completing the baseline questionnaire, another 337 patients (20%) dropped out for various reasons, including declining health or death. A total of 1103 (65%) patients responded to the baseline questionnaire of the eQuipe study.

### Measures

Perceptions of ACP involvement were assessed by three statements, developed by Rietjens et al. [6]: “I feel I am as much involved as I want to be in decisions about my medical treatment and care,” “I feel my immediate family and friends know what my preferences are regarding my future medical treatment and care,” and “I feel my physicians know what my preferences are regarding my future medical treatment and care,” all rated on a 5-point Likert scale. Emotional functioning was measured by the EORTC QLQ-C30. Symptom burden was assessed using the number of clinically important symptoms (pain, fatigue, nausea, vomiting, dyspnea, insomnia, appetite loss, constipation, and diarrhea) of the EORTC-QLQ-C30 using the thresholds of Giesinger et al. [7].

#### Demographic and clinical data

Age, gender, marital status, migrant background, and education were self-administered. Comorbidity in the past 12 months was assessed with the adapted self-administered comorbidity questionnaire. Primary cancer type and date of diagnosis were obtained from the Netherlands Cancer Registry.

### Statistical analysis

Descriptive statistics were used for sociodemographic and clinical characteristics and the ACP perceptions. Responses to the ACP statements were recategorized into (strongly) disagree (1–2), neutral (3), and (strongly) agree (4–5). Differences in emotional functioning between these groups were determined using a one-way ANOVA. The three statements were combined in an ACP mean score that was linearly transformed to a 0–100 ACP scale, a higher score implying more perceived involvement in ACP [6]. The three statements had a Cronbach alpha of 0.79. Multivariable linear regression analysis using forced entry was performed to assess the association between the ACP scale and emotional functioning while adjusting for the confounders gender, age, migrant background, education, marital status, and symptom burden. All statistical analyses were performed using STATA version 16, and a two-sided significance level of *p*<0.05 was used.

## Results

In total, 1001 (91%) completed the ACP statements of the baseline questionnaire. Fifty percent were male, and the mean age was 65 years (SD 9.8). Most patients were diagnosed with lung cancer (30%), breast cancer (16%), or colorectal cancer (15%). The majority (74%) received treatment in the prior 3 months. Two-third reported one or more comorbidities (67%) (Table 1).

**Table 1:** Sociodemographic and clinical characteristics of patients with advanced cancer (*n*=1.001)

		% (*n*)
Age	Mean (SD)	65 (9.8)
Gender	Male	50 (502)
	Female	50 (499)
Marital status	Partner	83 (830)
	No partner	17 (170)
Migrant background	No	97 (964)
	Yes	3.4 (34)
Education level^a^	Low	29 (284)
	Medium	43 (422)
	High	29 (285)
Primary tumor	Lung	30 (303)
	Breast	16 (163)
	Colorectal	15 (154)
	Prostate	12 (125)
	Other	26 (256)
Time since primary diagnosis	<1 year	32 (318)
	1–5 years	44 (443)
	>5 years	24 (240)
Treatment in the prior three months^b^	No treatment	26 (260)
	Chemotherapy	29 (287)
	Radiotherapy	7.0 (69)
	Surgery	20 (200)
	Immunotherapy	23 (223)
	Other	22 (225)
Number of comorbidities	0	33 (329)
	1	36 (356)
	>1	31 (303)
Number of severe symptoms^c^	Mean (SD)	2.3 (1.9)

<5% missings were omitted from the table

Due to missings, frequencies do not add up to the total number of patients

Due to rounding, percentages may not equal 100%

^a^Education according to the ISCED guidelines: low = none or primary school, medium= secondary school, high= (applied) university

^b^Treatment in the prior 3 months: more than one treatment is possible, so the percentages for the treatment modalities do not sum up to 100%

^c^According to the thresholds of clinical importance of Giesinger et al. [7]

Most patients (87%) felt as much involved in decisions about their *future* medical treatment and care as they wanted to be. Most patients felt that their family and friends (81%) and physicians (75%) were aware of these preferences. A minority of patients felt not involved in decision-making about future medical treatment and care (2.7%) and felt that their family and friends (5.7%) and physicians (7.7%) were not aware of these preferences either (Table 2). The ACP scale had a mean of 75 (SD 17).

**Table 2:** Perceived involvement of patients with advanced cancer regarding three aspects of ACP and mean emotional functioning (*n*=1.001)

Aspect of ACP		% (*n*)	Emotional functioning (mean (SD))
Patients’ involvement	(Strongly) agree	87 (873)	79.1 (20.6)*
	Neutral	10 (101)	74.5 (21.4)
	(Strongly) disagree	2.7 (27)	73.8 (20.4)
Immediate family and friends’ involvement	(Strongly) agree	81 (815)	79.6 (20.0)*
	Neutral	13 (129)	74.5 (22.6)
	(Strongly) disagree	5.7 (57)	72.2 (23.7)
Physicians’ involvement	(Strongly) agree	75 (754)	79.7 (20.2)*
	Neutral	17 (170)	74.8 (22.3)
	(Strongly) disagree	7.7 (77)	75.4 (21.2)

<5% missings were omitted from the table

*p<0.05 tested by one-way ANOVA

Emotional functioning mean scores differed significantly between patients who (strongly) agreed with “being involved in future decision-making as much as I want,” neutral patients, and patients who (strongly) disagreed, respectively, 79, 75, and 74. Emotional functioning scores also differed significantly for the statements regarding the involvement of family and friends and physicians (Table 2).

The multivariable linear regression analysis showed a positive association (*b*=0.162, *p<0.001*, 95%CI[0.095;0.229]) between the patients’ perceptions of ACP involvement and their emotional functioning while taking gender, age, migrant background, education, marital status, and symptom burden into account.

## Discussion

Most patients with advanced cancer felt involved in decisions about their *future* medical treatment and care. According to most patients, their immediate family and friends, and physicians were aware of their preferences for *future* medical treatment and care. Patients’ perceptions of ACP involvement and their emotional functioning were positively associated.

The positive association between ACP perceptions and emotional functioning confirms the results of a systematic literature review showing that ACP (actual involvement) is positively associated with psychosocial measures such as stress, anxiety, and depression [2]. Whether ACP actually positively affects emotional functioning or (vice versa) better emotional functioning leads to better communication about future care planning cannot be determined on the basis of our study and the literature. However, a recent European cluster-randomized trial among patients with advanced cancer found no effect of an ACP intervention on emotional functioning [3]. This might be due to the standardization of the ACP intervention in a research context, unable to adapt to local circumstances and needs [3]. Furthermore, the positive association found in our study was small. Although statistically significant differences in emotional functioning were found between patients with different ACP perceptions, these differences may not be clinically relevant [8]. Apart from the positive association between ACP and emotional functioning, ACP is also positively associated with receiving appropriate end-of-life care, increased satisfaction with care, decreased use of hospital care, and increased use of hospice care [2].

### Strengths and limitations

A major strength of this study is the large cohort of patients, linked to the National Cancer Registry for clinical information. However, selection bias is likely to be present as patients were mainly recruited by their own physicians. This influences the representativity of the study population as they are likely to have a relatively better health. Moreover, we did not include all hospitals of the Netherlands. In addition, patients’ involvement in ACP may be overestimated due to the underrepresentation of patients with a migrant background. These patients are less likely to be involved in ACP than patients without a migrant background [9]. Moreover, the ACP statements used were self-developed and not validated [6]. Lastly, although a statistical relationship has been found, because of the cross-sectional analyses of baseline data, no causal relationship has been investigated.

### Practical implications and future research

Patients who feel involved in decisions about *future* medical treatment and care and feel that their immediate family, friends, and physicians are also involved report better emotional functioning. Patients seem open to ACP as the majority reported to have discussed their preferences with their immediate family and friends. Physicians could play an active role in discussions of patients’ wishes and preferences regarding future medical treatment and care. Especially as it is known that patients, their family members and physicians experience barriers to initiate ACP, including fear to provoke distress and disrupt hope [10]. Future studies should further investigate the causal relationship between ACP perceptions and emotional functioning.

## Conclusion

This study shows that the majority of patients with advanced cancer in the Netherlands feel involved in decisions about *future* treatment and care and that their family, friends, and physicians are aware of their preferences for *future* treatment and care. Only a limited number of patients feel not to be involved in ACP. Perceptions regarding ACP involvement and emotional functioning are positively associated. This positive association, although small, underpins the importance of further research into the causal relationship between ACP and emotional functioning.

## Data Availability

Since 2011, PROFILES registry data is freely available according to the FAIR (Findable, Accessible, Interoperable, Reusable) data principles for non-commercial (international) scientific research, subject only to privacy and confidentiality restrictions. The datasets analyzed during the current study are available through Questacy (DDI 3.x XML) and can be accessed by our website (www.profilesregistry.nl). In order to arrange optimal long-term data warehousing and dissemination, we follow the quality guidelines that are formulated in the “Data Seal of Approval” (www.datasealofapproval.org) document, developed by Data Archiving and Networked Services (DANS). The data reported in this manuscript will be made available when the eQuiPe study is completed.
